# The Time Course of Inhibition of Return: Evidence from Steady-State Visual Evoked Potentials

**DOI:** 10.3389/fpsyg.2017.01562

**Published:** 2017-09-12

**Authors:** Ai-Su Li, Gong-Liang Zhang, Cheng-Guo Miao, Shuang Wang, Ming Zhang, Yang Zhang

**Affiliations:** Department of Psychology, Soochow University Suzhou, China

**Keywords:** inhibition of return, steady-state visual evoked potentials, time course, purely evaluating of inhibition of return, shifts of exogenous attention

## Abstract

Inhibition of return (IOR) refers to slower responses to targets at a previously cued location than that at an uncued location. The time course of IOR has long been a topic of interest in the field. Investigations into the time course of IOR are typically performed by examining the magnitude of IOR under various cue-target onset asynchrony (CTOA) conditions. Therefore, the results are vulnerable to influence of factors that could affect the target processes (e.g., the frequency of the target type). In the present study, steady-state visual evoked potentials (SSVEPs) were implemented to directly take a continuous measurement of the degree to which cued location is processed, eliminating the influence mentioned above. The results indicate that, relative to the baseline interval (−400 to 0 ms), the presence of peripheral cues generated a typical two-stage effect on the SSVEP amplitude evoked by a 20 Hz flicker. Specifically, after the onset of the peripheral cues, the SSVEP amplitude first showed a significant increase, which subsequently turned into a significant inhibition effect after 200 ms. These results provide a continuous time course diagram of the cueing effect and suggest an effective way for future investigations of controlling the masking effects of target stimuli processing on IOR.

## Introduction

The ability to efficiently search for a particular object or target (e.g., looking for a friend in a busy train station) in a cluttered environment is a fundamental skill of the human cognitive system (Najemnik and Geisler, 2005). To maintain search efficiency, the cognitive system must reduce the probability of returning to previously searched locations (Klein, 1988). Previous studies have demonstrated that inhibition of return (IOR) may involve a mechanism supporting optimized search efficiency by discouraging attention from returning to a previously searched location (Macinnes and Klein, 2003; MacInnes et al., 2014).

IOR was initially revealed by Posner and Cohen (1984), who found that approximately 250 ms following an uninformative exogenous cue, the response of participants was slower to targets at cued locations than to targets at uncued locations. Researchers have referred to this suppression effect as IOR and have conducted extensive research on it, including its time course (Lupiáñez et al., 2001; McCrae and Abrams, 2001; Samuel and Kat, 2003; Müller, 2008), components (Chica et al., 2010; Hilchey et al., 2012, 2014), mechanism (Fuentes et al., 2000; Satel and Wang, 2012; Zhang et al., 2013), plasticity (Xu et al., 2015), as well as electrophysiological correlations (Prime and Ward, 2006; Prime and Jolicoeur, 2009; Satel et al., 2013, 2014a).

The time course of IOR is one of the hottest subjects in the field of IOR (Lupiáñez and Weaver, 1998; Tassinari et al., 1998; Lupiáñez et al., 2001; Pratt and Hirshhorn, 2003; Samuel and Kat, 2003; Funes et al., 2005; Hickey et al., 2009). Most studies examine the time course of IOR by investigating the magnitudes of IOR under different cue-target onset asynchrony (CTOA, i.e., the time interval between cue and target onset) conditions. However, it is noteworthy that this type of design has two intrinsic shortcomings. First, it depends on a comparison of differences between cued and uncued conditions in the processing of the targets, which itself requires the input of attentional resources and thus might mask IOR effects to a certain extent. In other words, it infers the time course of IOR indirectly from investigating the interaction effect between IOR and target processing. Hence, theoretically, any difference in the time course of IOR may merely reflect different sensitivities to IOR with regard to the various cognitive processes involved in different tasks (e.g., detection vs. discrimination tasks). Second, theoretically, an unlimited number of CTOA settings could be included to fine-tune the investigation of the time course of IOR. However, limited by efficiency and time, there are few studies including more than 10 CTOA settings. Indeed, a design involving more than 10 experimental conditions represents a considerable challenge regarding time and effort for both researchers and participants. To date, there is only one study that uses more than 10 CTOAs, at the expense of less than around 20 trials per condition (46 CTOAs; Song et al., 2014).

An ideal measurement technique, to avoid the shortcomings mentioned above, should satisfy two requirements. First, the measurement index should be independent of target processing, i.e., it should not rely on the processing of targets so that the effects of target-triggered attention (or cognitive resources) can be eliminated and the IOR effects can be directly measured. Second, a measurement that has high time-precision but is not a simple probing of several or even several tens of dispersed time points should be performed on the time sampling. An electrophysiological technique called steady-state visual evoked potentials (SSVEPs) has been proven to be a useful tool for satisfying those two requirements (Müller et al., 1998, 2016; Keitel and Müller, 2016). SSVEPs refers to a continuous and periodic potential response of the extrastriate cortex to a flickering stimulus and has the same temporal frequency as the driving stimulus (Norcia et al., 2015). The spontaneous property of SSVEPs enables us to investigate IOR effects without requiring the subject's response to the flickering stimuli. Although, many transient visual event related potential components (P1/N1, Nd) have also been demonstrated to be sensitive to IOR (Prime and Ward, 2006; Prime and Jolicoeur, 2009; Satel et al., 2013, 2014a; Xu et al., 2016), the SSVEPs has advantages over visually evoked potentials. It is an ongoing waveform that could enable us to estimate the time course of IOR continuously.

With the SSVEPs technique, researchers have successfully conducted a large number of studies on the time course of endogenous spatial and feature-based attention (Müller et al., 1998; Müller, 2008; Andersen and Müller, 2010; Andersen et al., 2015). For example, Müller et al. (1998) utilized SSVEPs to track the time course of the attention deployment, demonstrating a close temporal relationship between the amplitude of SSVEPs and the shift of endogenous attention. Specifically, they found that around 250–300 ms after an endogenous cue, the magnitude of SSVEP elicited by the attended flicker increased significantly from the baseline period.

**Figure 1:**
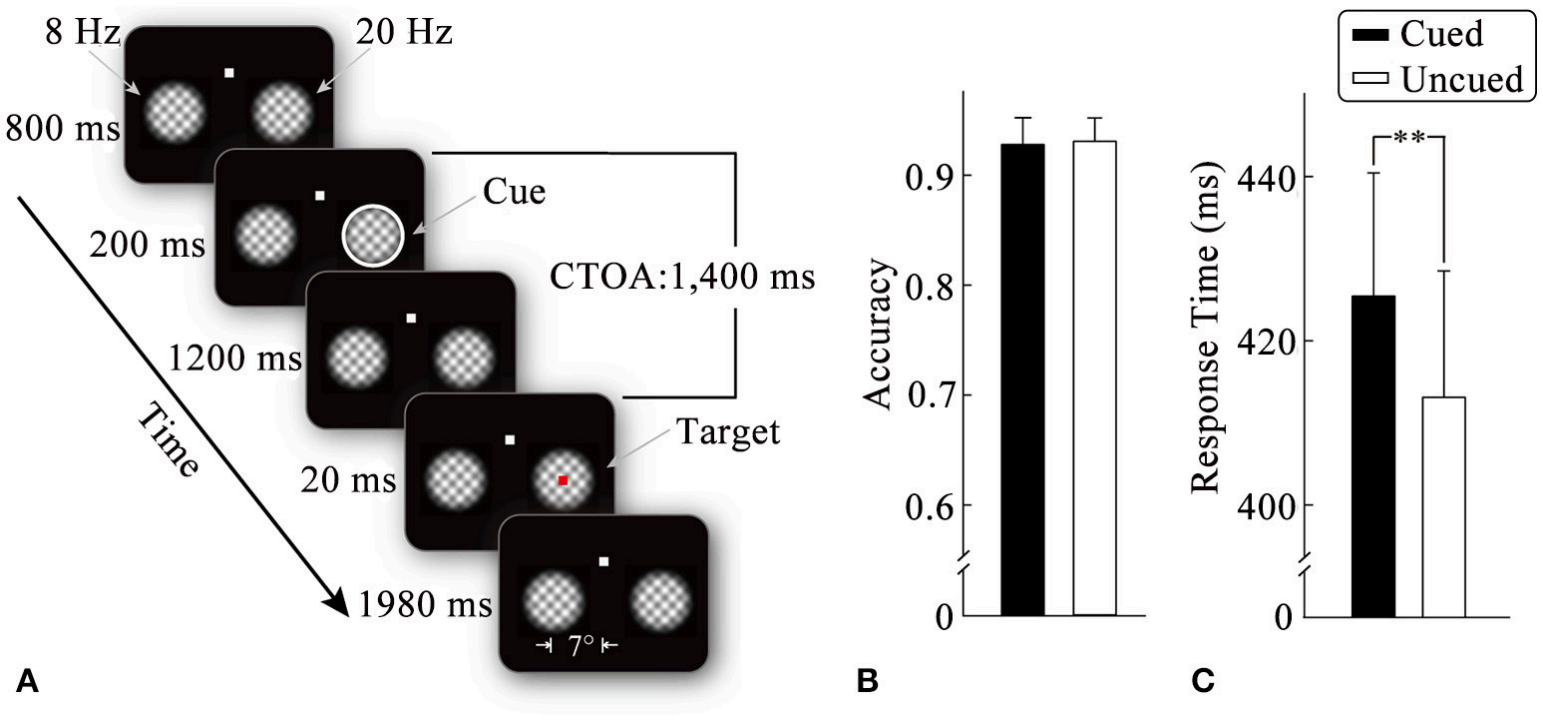
Stimulus display sequences in an example trial **(A)**. Mean detection scores **(B)** and response times **(C)** for target stimulus as a function of the cueing condition. Standard error bars are shown. ^**^p < 0.01.

In contrast, few studies have investigated IOR with SSVEPs. As far as we know, there is only one “pilot-type” study examine the time course of IOR using SSVEPs (Satel et al., 2014b). Although Müller et al. (1998) and Müller (2008) have successfully used SSVEPs to track the time course of endogenous attention, previous studies have demonstrated that endogenous attention and IOR are independent of each other (Lupiáñez et al., 2004, 2012; Martín-Arévalo et al., 2006; Chica and Lupiáñez, 2009). Therefore, it is still unclear whether SSVEPs are sensitive to IOR. In the present study, we try to use the SSVEP technique to track the time course of IOR with high time precision. Specifically, we used two task-irrelevant stimuli, to which no response is required, flickering at different frequencies to tag the two spatial locations (Figure 1A). Referring to the time dependence of the induced SSVEP amplitude, we investigate the time course of IOR effects triggered by peripheral cues. If the SSVEP signal is sufficiently sensitive to IOR, the experimental results are expected to be consistent with the hypothesis that IOR effects occur at approximately 250 ms. Additionally, the SSVEP amplitude is expected to follow a change pattern (IOR) in which it first appears to increase temporarily after the presentation of a peripheral cue, which would capture the attention, and then subsequently drops down below the baseline. Conversely, if SSVEP is only sensitive to early attention processing and is insensitive to IOR, then SSVEP amplitude is expected to only increase in the early stage after the presence of a peripheral cue.

## Methods

### Participants

Nineteen healthy volunteers (14 females, 18~26 years old) of Soochow University, naive to the purpose of the study, participated in the experiment for financial compensation. All participants, with normal or corrected-to-normal vision, had never participated in any similar experiment. Two participants were excluded due to a low signal-to-noise ratio of the SSVEP (SNR<1). All subjects gave written informed consent by the Declaration of Helsinki. The study was approved by the Academic Committee of the College of Education, Soochow University.

### Stimuli and procedure

The experiment, generated in Matlab using Psychtoolbox (Kleiner et al., 2007), was run on a computer with the Ubuntu14.04 operating system. Stimuli driven by a NVidia GT630 graphics card were presented against a black background on a 22-inch CRT monitor (Philip 202P40; resolution: 1,024 × 768 pixels) at a refresh rate of 120 Hz. The participants sat in a dimly lit room, viewing the screen from a distance of 80 cm; they were instructed to maintain fixation on the fixation point throughout the experiment. The behavioral response times (RTs) were collected via a 7-key gamepad (Microsoft X04-97602). The stimuli were grid gratings with luminance changes sinusoidal at a frequency of 8 Hz and 20 Hz at left and right locations, respectively. Those two frequencies were chosen because they elicited high SNRs in a pilot experiment with one participant who did not participate in the main experiment.

An example of the stimulus display and trial sequence is illustrated in Figure 1A. Each trial began with the presentation of a fixation display, consisting of a white fixation point (0.3° × 0.3°) and two plaid gratings (4° × 4°). The plaid gratings were composed of two orthogonal gratings (spatial frequency: 1.2 cycles/degree) multiplied by a 2D tapered cosine envelope (Tukey window). The fixation point was centered 4° above the center of the screen, whereas the two grid gratings were centered 7° to the left and right of the screen center. The luminance of the plaid gratings changed periodically (from left to right: at a frequency of 8 and 20 Hz, respectively) to generate flickers until the end of the trial. (2) After an 800-ms-fixation interval, the peripheral cue (a white annulus, thickness: 0.27°) was equally likely to appear at either of the two flicker stimuli (50% validity) for 200 ms. (3) After the peripheral cue had disappeared for 1,200 ms, the target stimulus (a red square: 0.3° × 0.3°) was presented randomly in one of the flicker stimuli with equal probability for 20 ms. The participants were required to detect the target by pressing a pre-specified key as quickly and accurately as possible. No target was presented and no response required on 20% of the trials, which served as catch trials to minimize anticipatory responding on the remaining trials. (4) After the offset of the target (1,980 ms), the two flickers disappeared, which signified the end of one trial. Before the experiment, the participants were informed that the cue was unpredictable of the target location.

The experiment consisted of 10~20 practice trials, followed by 10 blocks of 40 trials each, for a total of 400 trials. Each block was approximately 4 min long, containing 16 cued location trials, 16 uncued location trials, and 8 catch trials. Break time between blocks was controlled by participants. It is noteworthy that the flicker stimuli presented in the lower visual field would better activate the upper area of the early visual cortex (Dumoulin and Wandell, 2008), which could make us better record SSVEP signals via the parietal electrodes.

### Electrophysiological recording

A Quick-Cap with 64-channel electrodes was used to collect the electroencephalogram (EEG) data using Neuroscan software (SCAN 4.3) according to the 10–20 system. The electrooculogram (EOG) activity was recorded both vertically, from two bipolar electrodes positioned 1.5 cm above and below the left eye (vEOG), and horizontally, from electrodes placed 1.5 cm lateral to the outer canthi of both eyes (hEOG). EEG and EOG were sampled continuously at a rate of 1,000 Hz, amplified and filtered by Synamps 2 amplifiers (0.05~100 Hz band-pass). The recording was referenced to the left mastoid, during which the prefrontal electrodes were grounded. Electrode impedances were kept less than 5 kΩ.

After manually excluding the apparent artifacts, ocular artifacts were corrected by a regression algorithm (Gratton et al., 1983). The EEG data were segmented into epochs starting 400 ms before, and 1,400 ms after the onset of the peripheral cue^1^ and then corrected by a linear detrend algorithm to eliminate the influence of linear drift. The baseline was corrected by subtracting the mean of the signals within a time window of −400 to 0 ms (the peripheral cue onset). Trials with artifacts whose amplitudes exceeded ±75 μV in any of the EEG channels were rejected, followed by removing those with eye movements or eye-blinks using a peak-to-peak moving window approach. Specifically, epochs in either of the EOG channels containing peak-to-peak amplitudes above a threshold of ±50 μV within a 100 ms moving window that slides at a step of 4 ms were rejected (Luck, 2014). The remaining trials without response errors and artifacts were re-referenced to an average of left and right mastoids. After that, ERP waves were calculated separately for each condition at all electrodes time-locked to the onset of the peripheral cues. Overall, approximately 25% of the data were discarded (around 6, 7, and 12% for response errors, eye movements/blinks and other types of artifacts such as the muscle or movement artifacts, respectively), with about 149 trials remaining for each condition.

## Data analysis and results

### Behavioral data

For each condition of each participant, a non-recursive trimming procedure was used to discard the extreme outliers of correct response times (Selst and Jolicoeur, 1994). Two-tailed paired *t*-tests were used to assess the difference between the cued and uncued trials (without response errors and outliers) in accuracy and RTs. Overall, false alarms (responses to catch trials) accounted for less than 3% of the catch trials in the experiment.

#### Accuracy

Figure 1B illustrates the mean detection scores under the cued and uncued conditions. Two-tailed paired *t*-tests did not show significant effects of the cueing on accuracy (*t* < 1).

#### Response time

Mean response times as a function of the cueing condition are shown in Figure 1C. Two-tailed paired *t*-tests revealed that correct RTs without outliers were significantly faster (*t*_16_ = 3.04, *p* < 0.01, η^2^ = 0.37) for the uncued condition (414 ms) than for the cued condition (427 ms), suggesting a typical IOR effect.

### Event-related potential (ERP) evoked by the peripheral cues

**Figure 2:**
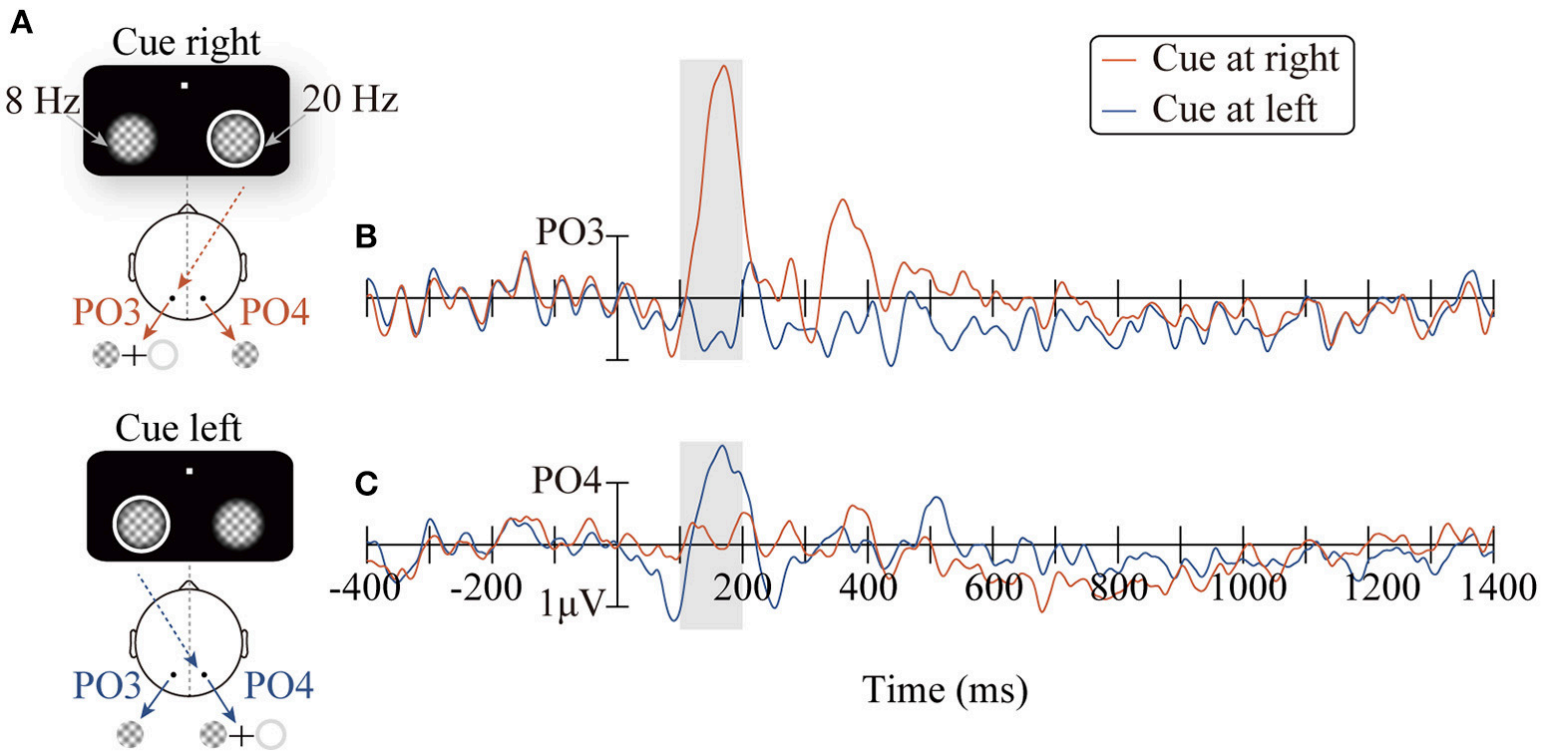
The diagrams of the two conditions: CR and CL for presenting the peripheral cue at the right (red line) and the left location (blue line), respectively **(A)**. ERPs waveforms time-locked to the peripheral cue at the PO3/PO4 electrodes **(B,C)**.

Figure 2 shows the ERP waveforms (PO3/PO4) elicited by the peripheral cues. Consistent with the retinal mapping, stimuli presented contralateral to the PO3/PO4 electrodes evoked greater amplitudes than stimuli presented ipsilateral to the PO3/PO4 electrodes within the time window of 100–200 ms (Wandell et al., 2007). Specifically, the right cue (appeared at the location of the 20 Hz flicker), compared to the left cue (appeared at the location of the 8 Hz flicker), elicited higher amplitudes on the PO3 electrode in the left occipitoparietal area (Figure 2B, *t*_16_ = 5.59, *p* < 0.001). The left cue, compared to the right cue, elicited larger amplitude on the PO4 electrode, which was located in the right occipitoparietal area (Figure 2C, *t*_16_ = −2.15, *p* < 0.05).

### SSVEP

#### Signal-to-noise ratio (SNR)

Before the statistical analysis, we first analyzed the SNR of SSVEPs elicited by 8 Hz and 20 Hz flicker stimuli separately. A fast Fourier transform (FFT) was applied to the ERPs waveforms of each participant. Then, a specified frequency point was labeled *i*, and the mean amplitude of three frequency points (*i*–4, *i*–3, *i*–2 and *i*+2, *i*+3, and *i*+4) before and after *the ith frequency point* served as the noise level to estimate the SNR of the corresponding SSVEP signal. By comparing the level of the desired signal to the level of background noise, we can estimate the SNR of the specified frequency (20 and 8 Hz).

**Figure 3:**
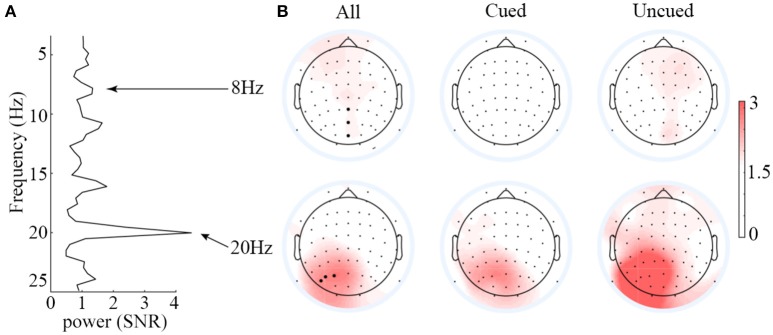
Averaged SNR spectrum of the PO3, PO5, and PO7 electrodes **(A)** and scalp topographies of the SSVEPs elicited by 8 and 20 Hz flickers **(B)**. Overall, the SNR of 8-Hz-flicker-elicited SSVEPs was very low, and it is hard to differentiate the signal from noise; the SNR of 20-Hz-flicker-elicited SSVEPs was relatively higher over the left occipitoparietal scalp.

Figure 3 presents the scalp topographies of SNR for 8 and 20 Hz SSVEPs. Overall, the SNR for 8 Hz was much less than that for 20 Hz. The average SNR of the three strongest signal electrodes for 8 Hz SSVEPs was 1.34, whereas that for 20 Hz SSVEPs was 4.49. Hence, only the 20 Hz SSVEPs amplitude was selected and submitted to statistical analysis. Based on this criterion, the data from two participants (SNR<1, meaning signal and noise could not be differentiated from each other) were excluded. Analyses of the SSVEP amplitudes were performed on electrode PO3, PO5, and PO7. The three electrodes were selected based on the average SNR of the 20 Hz SSVEPs across all conditions to avoid the statistical circularity.

#### SSVEP amplitudes of 20 Hz flicker stimulus

**Figure 4:**
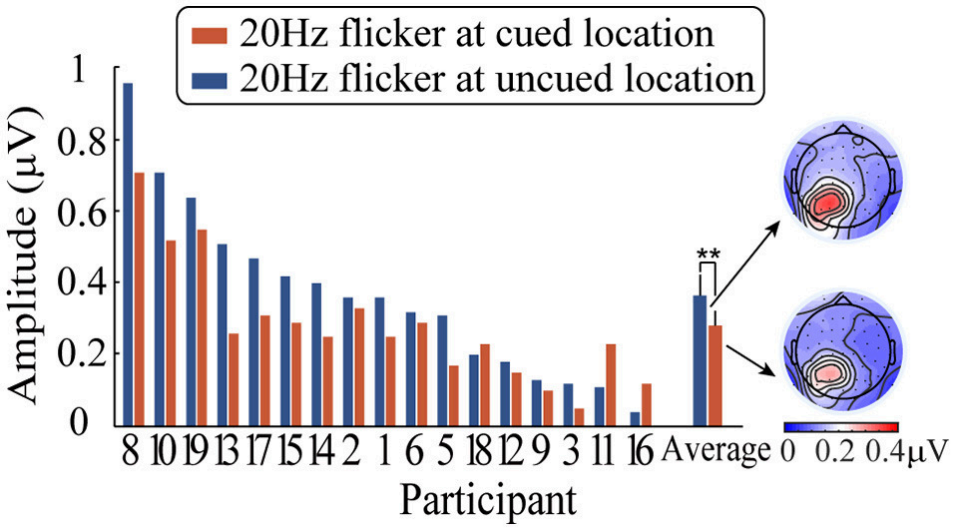
SSVEP amplitudes elicited by a 20 Hz flicker stimulus when it was cued (blue) and uncued (red) as well as the corresponding topographic maps. ^**^ indicates *p* < 0.01.

After averaging, a fast Fourier transform (FFT) was applied to the ERPs waveforms for a time window from 0 to 1,400 ms after the peripheral cue onset, followed by computing the SSVEP amplitude of the 20-Hz-flicker stimulus (i.e., the square root of the SSVEP power). Subsequently, the SSVEP amplitude was submitted to a 2 (relative location of the 20 Hz flicker: at the cued vs. uncued locations) × 3 (electrodes: PO3, PO5, and PO7) repeated-measures analysis of variance (ANOVA). Figure 4 shows the SSVEP amplitudes elicited by the 20 Hz flicker stimulus during the period from the peripheral cue onset to the presence of the target stimulus for each participant. Only the main effect of the relative location of the 20 Hz flicker was significant, *F*_(1, 16)_ = 11.10, *p* < 0.01, η^2^_*p*_ = 0.41. The 20 Hz flicker evoked larger SSVEPs at the uncued locations (0.37 μV) than that at the cued locations (0.28 μV), suggesting a significant IOR effect. Neither the main effect of the electrodes (*p* > 0.12) nor the interaction between the relative location of the 20 Hz flicker and the electrodes (*p* > 0.55) reached significance.

#### Analysis and results of the SSVEPs

(1) $$\begin{array}{l} Amplitude_{n}\left(\text{k}\right)=\\ \sqrt{{\left(\frac{\sum_{k=0}^{n}\lambda^{n-k}y(k)sin\;(\frac{2\pi kf_{0}}{f_{s}})}{\sum_{k=0}^{n}\lambda^{n-k}sin^{2}\;(\frac{2\pi kf_{0}}{f_{s}})}\right)}^{2}+{\left(\frac{\sum_{k=0}^{n}\lambda^{n-k}y(k)cos\;(\frac{2\pi kf_{0}}{f_{s}})}{\sum_{k=0}^{n}\lambda^{n-k}cos^{2}\;(\frac{2\pi kf_{0}}{f_{s}})}\right)}^{2}} \end{array}$$

**Figure 5:**
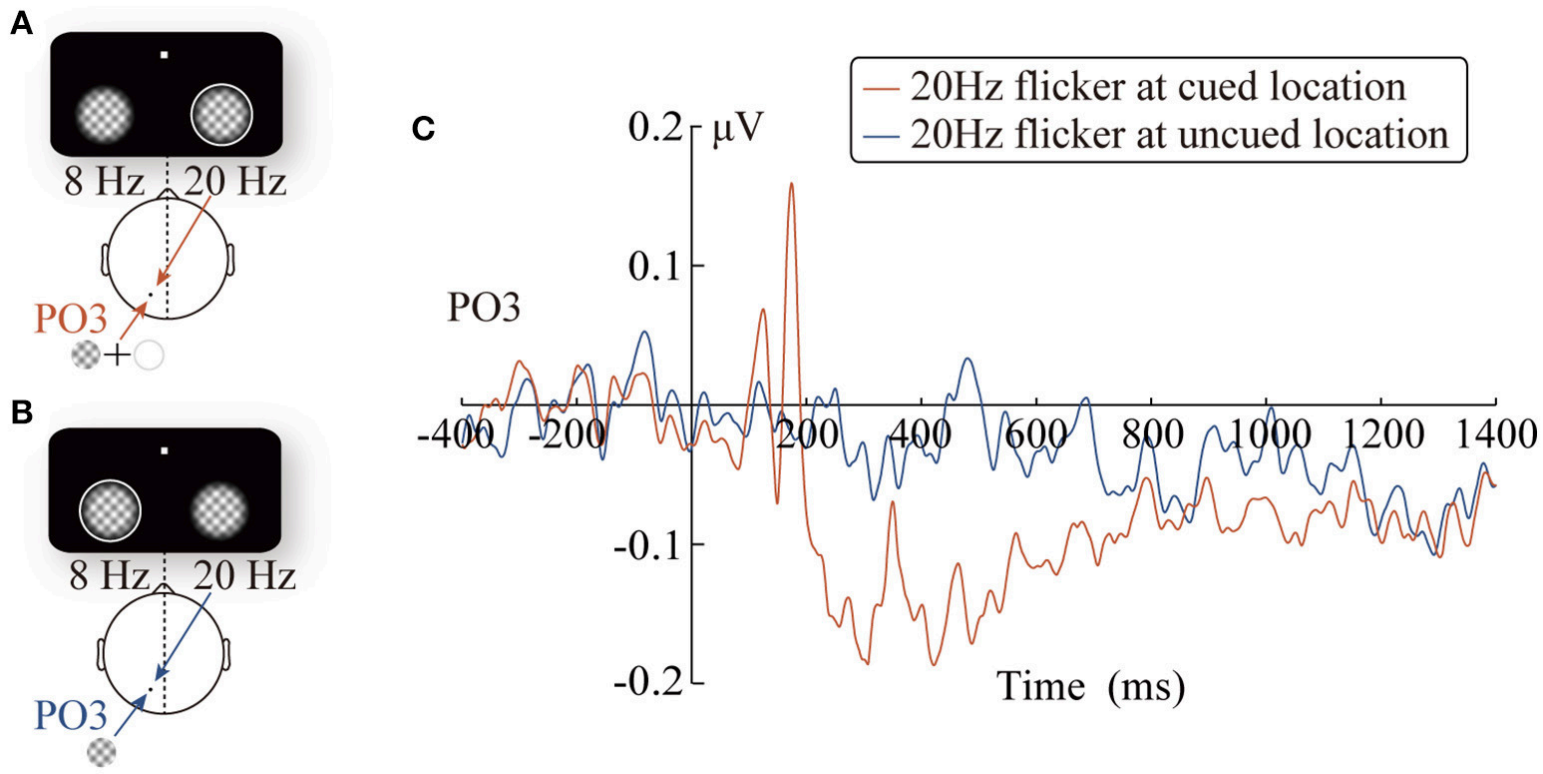
The diagrams of the two experimental conditions: the 20 Hz flicker was cued **(A)** and uncued **(B)**. Grand-average SSVEP waveforms at the PO3 electrode elicited by the 20 Hz flicker stimulus **(C)**.

The amplitude of the SSVEP signals was extracted using an adaptive filter proposed by Tang and Norcia (1995) based on the recursive least squares method. Specifically, the amplitudes of the SSVEP signal were estimated by Equation (1). The *y*(κ), *f*_0_, and *f*_*s*_ in Equation (1) indicate the kth sample, the stimulus frequency, and the sampling rate respectively. The index n of “Amplitude”_*n*_(k) implies that the most recent data used to estimate “Amplitude”_*n*_(k) is y(n). The λ is a constant and is always set to larger or equal to 0.995 (see Tang and Norcia, 1995; for more detail). Compared to the sliding-window filter based on the discrete Fourier transform (Andersen and Müller, 2010), the adaptive filter has higher efficiency and better anti-interference ability, and it has been successfully applied in previous studies involving SSVEP signal extraction (Tang and Norcia, 1995; Zhang et al., 2011). Then, a baseline correction was performed in the –400~0 ms period preceding the onset of the peripheral cue. Finally, based on the experimental hypothesis and on inspection of the waveforms of SSVEP, we performed the statistical analysis of the data for periods of 170~180 ms, 201~800 ms, and 801~1,400 ms (Figure 5C).

Taking the 400-ms precue SSVEPs elicited by the 20 Hz flicker as a reference, we analyzed the change pattern of the SSVEP amplitude after the cue onset in three time windows. More specifically, the mean SSVEP amplitude evoked by the cued 20 Hz flicker stimulus during three time windows was separately submitted into a two-tailed one-sample *t*-test. In other words, we evaluated whether the 20 Hz flicker SSVEP amplitudes in three time windows were significantly different from that in the baseline period. The results revealed a significant two-stage phenomenon after the presence of the peripheral cue: during a short period (170~180 ms) after the peripheral cue onset, the SSVEP amplitude elicited by the cued 20 Hz flicker was enhanced (*t*_16_ = 2.70, *p* = 0.017, η^2^ = 0.31); however, this facilitation effect soon turned into an inhibitory effect (201~800 ms: *t*_16_ = −2.57, *p* < 0.05, $\eta_{p}^{2}=$ 0.29; 801~1,400 ms: *t*_16_ = −2.91, *p* = 0.01, η^2^ = 0.35).

Comparing the difference in the SSVEP amplitudes evoked by the 20 Hz flicker at the cued and uncued locations, we investigated the impact of the peripheral cue on the SSVEP amplitude. The SSVEP amplitudes elicited by the 20 Hz flicker stimulus were submitted to a 2 (the relative location of the 20 Hz flicker: at cued vs. at uncued locations) × 3 (time windows: 170~180, 201~800, and 801~1,400 ms) × 3 (electrodes: PO3, PO5, and PO7) repeated-measures ANOVA. The *p*-values were adjusted by the Greenhouse–Geisser adjustment whenever appropriate and were reported as *p*_*c*_.

The main effect of the time windows was significant, *F*_(2, 32)_ = 21.74, *p*_*c*_ < 0.001, $\eta_{\text{p}}^{2}=$ 0.58, and the SSVEP amplitude showed a decreasing trend as time passed (the linear effect was significant, *F*_(1, 16)_ = 24.39, *p*_c_ < 0.001, $\eta_{\text{p}}^{2}=$ 0.60; the SSVEP amplitudes in the three time windows were 0.06 μV, –0.07 μV, and –0.07 μV, respectively). More importantly, the interaction between the relative location of the 20 Hz flicker and the time windows was significant, *F*_(2, 32)_ = 10.41, *p*_*c*_ = 0.001, $\eta_{p}^{2}=$ 0.39. The SSVEP amplitude evoked by the cued 20 Hz flicker showed a significant two-stage trend. During the early stage (170~180 ms), the SSVEP amplitude for the cued 20 Hz flicker was enhanced by the peripheral cue (0.15 μV), compared to that for the uncued 20 Hz flicker (−0.02 μV), *F*_(1, 16)_ = 4.97, *p* < 0.05, $\eta_{p}^{2}=$ 0.24. In other words, there was a significant facilitation effect in the early stage, which soon (200~800 ms) turned into a significant inhibitory effect, *F*_(1, 16)_ = 4.71, *p* < 0.05, $\eta_{p}^{2}=$ 0.23, i.e., the SSVEP amplitude evoked by the cued (−0.12 μV) but not the uncued (−0.02 μV) 20 Hz flicker decreased. This inhibitory effect, however, did not exist in the later stage (801~1,400 ms) after the cue presentation (*F* < 1). Consistent with this results, the changes of the inhibitory effect across time showed a significant cubic trend (A three-order polynomial curve fitted well with the difference wave during the time window of 200–1,400 ms, adjusted *R*^2^ = 0.63, *p* < 0.001). No other main effects or interactions were significant.

## Discussion

### Effects of IOR on the SSVEP amplitude

Since Müller and colleagues first utilized the SSVEP technique to investigate spatial attention, researchers have extended its use to various studies on endogenous attention and feature-based attention (Andersen et al., 2008, 2015; Robertson et al., 2012). However, few studies have been conducted to investigate IOR with SSVEP technique. Thus, whether SSVEPs are also sensitive to IOR remains an open question. We found that, compared to the control condition (the peripheral cue was presented contralateral to the 20 Hz flickering stimulus), the peripheral cue presented ipsilateral to the 20 Hz flicker stimulus significantly reduced the SSVEP amplitude elicited by the 20 Hz flickering stimulus (Figure 4). This result extends previous SSVEP findings suggesting that flicker-evoked SSVEPs are sensitive not only to endogenous attention but also to IOR. As found in previous studies of SSVEPs using functional imaging and source analysis (cortical current density), SSVEPs mainly originate from the early visual cortex, including V1, V3A, V4, and V5 (Müller et al., 2006; Di Russo et al., 2007). Müller and Kleinschmidt (2007) find that IOR can effectively influence the activation of the primary visual cortex (V1/2, V3/4); thus, the regulation of IOR on SSVEPs in the present study might stem from the modulation of IOR in the early visual cortex.

These results also have important implications for studies investigating the time course of IOR. As noted above, due to the indirectness of traditional measurement techniques, which evaluate the magnitude of IOR via the interaction between IOR and target processing, previous studies on the time course of IOR may be influenced by the different conditions in target processing (i.e., target stimulus processing could mask the IOR effect). Correspondingly, Lupiáñez et al. (2007) find that the moment when IOR appears behaviorally is subject to the characteristics of the target stimulus. Even in the same task, the behavioral response might show opposite cueing effects relative to the target stimulus at different frequencies. More specifically, a facilitation was triggered by a low-frequency stimulus, whereas IOR was triggered by a high-frequency stimulus under the same CTOA condition. Fortunately, we find that SSVEPs are also highly sensitive to IOR. Because task-irrelevant stimuli are used in SSVEPs that are not involved in the target processing, the problems caused by traditional indirect measurements can be avoided. Eliminating the influence of processing a task-relevant target on IOR, SSVEPs can be applied in future studies to investigate IOR better.

One might argue that since the 20 Hz is a second order harmonic of the alpha wave, it is likely that the results obtained on 20 Hz are contaminated by the alpha wave. The SNR data shown in the left panel of Figure 3, however, argue against this possibility. If the 20 Hz signal were originated from the alpha waves, we should expect to observe at least a comparable SNR between the alpha waves and the 20 Hz signal. From Figure 3, it is clear that the SNR of alpha waves (around 1) is far less than that of the 20 Hz (around 4), indicating that the 20 Hz signal is not contaminated by alpha waves.

### Time course of IOR

Traditionally, the time course of IOR has been inferred from the differences between cued and uncued conditions among various CTOA conditions (Lupiáñez et al., 1997; Samuel and Kat, 2003). However, the sampling rate of multi-setting CTOA studies is insufficient to provide an accurate depiction of the time course of IOR and might miss some meaningful information such as the peripheral cues evoked by oscillation (Song et al., 2014). With the SSVEP technique, IOR effects can be observed on a time scale of milliseconds, which may compensate the weakness of the traditional multi-setting CTOA method.

The statistical analysis of the time course provided a high time accuracy and continuous time mapping for the peripheral cueing effect. The SSVEP amplitude elicited by the 20 Hz flicker after the cue onset showed an apparent two-stage effect, compared to that during the 400 ms precue period. Approximately 180 ms after the presentation of the cue stimulus, there was a significant increase in the SSVEP amplitude (Figure 5C). However, this enhancement soon (after 200 ms) disappeared and turned into a significant inhibitory effect. The finding that the space facilitation triggered by the peripheral cue transformed into IOR at approximately 200 ms is consistent with previous behavioral studies. For instance, a meta-analysis of the IOR magnitudes over 27 studies by Samuel and Kat (2003) have suggested that facilitation turns into IOR at approximately 220 ms, which is in agreement with Klein's (2000) hypothesis regarding the time course of IOR.

Although the results obtained in the present study are consistent with those of previous research on the time course of IOR, it is noteworthy that the temporal patterns of the SSVEP amplitude (Figure 5C) were similar to traditional ERP waveforms. Therefore, it is possible that the results only reflect the inaccuracy in SSVEP signal extraction, but not the change in the SSVEP amplitudes. For example, the filter range was too broad to eliminate other frequency signals; thus, the 20 Hz SSVEP signal was not effectively extracted. Nevertheless, further analysis did not support this possibility: the SSVEP signal extraction method implemented in this study has been proven by a vast number of previous studies to be a highly efficient method for signal extraction (Norcia et al., 2015), and has also been successfully applied to extract the time course of SSVEP amplitudes. Zhang et al. (2001) use SSVEPs to label the changes in competing signals in binocular rivalry. More importantly, carefully comparing the cue-locked ERP waveforms (Figure 2B) and SSVEP amplitudes (Figure 5C), it is reasonable to rule out the possibility noted above. As observed in Figure 2B, the ERPs of the PO3 electrode (red line) evoked by the peripheral cue presented at the 20 Hz flicker location showed a greater amplitude than the baseline within the time window of 150~200 ms (*t*_16_ = 6.38, *p* < 0.001) and the subsequent 200~500 ms (*t*_16_ = 2.39, *p* < 0.05). If the SSVEP amplitude only reflected the ineffective filtering of ERP data, then the pattern of the 20 Hz SSVEP amplitude would be consistent with the EPR results, i.e., an amplitude greater than baseline in the 150~200 ms and 200~500 ms periods. Contrary to this expectation, the SSVEPs elicited by the 20 Hz flicker only showed greater amplitude in a short period (approximately 170~180 ms) after the cue onset and soon decreased below the baseline level (–400~0 ms) until the target stimulus was presented.

Interestingly, compared to the contralateral condition, the SSVEP amplitude elicited by the 20 Hz flicker stimulus presented ipsilateral to the cue showed a trend of early enhancement and subsequent inhibition; however, this trend was not significant in the 801~1,400 ms period. There are two possible explanations for this result. First, the control condition in which the 20 Hz flicker stimulus was presented contralateral to the cue may not be a suitable baseline for comparison because there were other stimuli (the cue) presented in it. In contrast, the 400-ms time window before the peripheral cue may be a more suitable baseline because, during that period, only the flicker stimuli were presented. Second, this pattern of results might suggest that SSVEPs only identify partial IOR in our study. Many studies have suggested that IOR might consist of two components: while an input/sensory adaptation based component occurs in the early stages of processing, an output/decision-based component takes place in the late stage of processing (Taylor and Klein, 2000; Hilchey et al., 2014). Compatible with the two components theory, numerous ERPs studies have demonstrated that IOR can not only express in the early P1/N1 component of the visual cortex (Prime and Ward, 2006; Prime and Jolicoeur, 2009; Satel et al., 2012, 2013, 2014a) but also manifest in the late response-selectivity components such as Nogo-N2 and the conflict-processing-related N450 (Prime and Ward, 2006; Tian and Yao, 2008; Zhang et al., 2012). Therefore, it is possible that the SSVEP signal from the visual cortex is only sensitive to the former input/sensory adaptation based component but not to the latter output/response decision-based component. This may explain why there was no significant difference in the SSVEP amplitudes between the two conditions (the cue presented at same vs. opposite side of the 20 Hz flicker stimulus) during 801~1,400 ms period. SSVEP signals are insensitive to the output based component, which mainly affects the late stages of processing (Hilchey et al., 2014), and thus showed no difference in the late stage. It is noteworthy that the current design cannot weight the probabilities of these two explanations and further research is needed to determine which explanation is more reasonable.

### Rapid shift of attention elicited by an exogenous spatial cue

The findings in this study are of great significance to the debated issue of whether peripheral cues speed up shift of spatial attention (Duncan et al., 1994). Numerous theories of spatial attention suggest that attention shift durations for endogenous and exogenous cues are different. Shifts of spatial attention initiated by endogenous cues involve multiple time-consuming processes, including discriminative processing of the cue information, disengagement, movement, and re-engagement of the attentional focus (Ward et al., 1996; Müller et al., 1998). In comparison, shifts of attention initiated by exogenous cues (i.e., the brightness change of the cue in this study) do not involve certain attention processing stages such as disengagement and re-engagement of the attentional focus so that they can be faster (Yantis, 1998). Nevertheless, these theoretical hypotheses lack electrophysiological evidence, and some researchers such as Müller (2008) have even found opposite results. In implementing SSVEP technology, Müller has found that the exogenous and the endogenous cues require a comparable amount of time (over 500 ms) to trigger a shift in spatial attention (Müller, 2008). It is noteworthy that the peripheral cue used by Müller (2008) was not a pure exogenous cue but carrying plentiful task-related information. The participants were asked to direct their attention to the flicker stimulus where the cue appeared. Therefore, there was no direct investigation of whether a pure exogenous cue that is uninformative about the target position can initiate a rapid shift of attention. Hence, an uninformative task-irrelevant peripheral cue was used with the SSVEP technique in the present study. The results reveal that compared to the baseline level, there was a significant increase in the amplitude of SSVEPs at approximately 200 ms after the presentation of the peripheral cue. This finding is consistent with the traditional hypothesis that exogenous cues can contribute to a faster shift of attention.

## Conclusion

1. SSVEPs are modulated not only by endogenous attention but also by IOR.
2. SSVEPs can serve as a valid measurement for purely evaluating the time course of IOR.
3. The shift of spatial attention initiated by a peripheral cue occurs approximately 200 ms after the presentation of a stimulus.

## Author contributions

YZ conceptualized and designed the study, AL, GZ, CM, and SW acquired the data, AL, GZ, SW, MZ, and YZ analyzed and interpreted the data, drafted the manuscript. AL and GZ contributed equally to the study and are co-first author.

### Conflict of interest statement

The authors declare that the research was conducted in the absence of any commercial or financial relationships that could be construed as a potential conflict of interest.

## Footnotes

^1^To avoid the startup transient of the adaptive filter used late to extract the SSVEP signal, the data were actually segmented into epochs starting from −800 to 1,600 ms after the onset of the peripheral cue. However, all of the artifact rejection and further analysis steps were carried out only on the interval between −400 to 1,400 ms.
